# For your eyes too: a new set of images of biological materials from the scientific commissioning of the MOGNO beamline at Sirius

**DOI:** 10.1107/S1600577525009993

**Published:** 2026-01-01

**Authors:** Marcos Vinicius Colaço, Thaina Alvarenga, Nathaly Lopes Archilha, Elis Barroso, Gustavo Colaço, Camila Cupello, Helio Ricardo da Silva, Gabriel Fidalgo, Anderson Marques Garcia, Samara Oliveira, Katrine Paiva, Gabriela Sena, Tayane Tanure, Regina Cely Barroso

**Affiliations:** a Physics Institute Universidade do Estado do Rio de Janeiro (UERJ) Rua São Francisco Xavier 524 Rio de Janeiro RJ 20550900 Brazil; b Brazilian Synchrotron Light Laboratory (LNLS) Brazilian Center for Research in Energy and Materials (CNPEM) Campinas São Paulo13083-970 Brazil; c Department of Archaeology Universidade do Estado do Rio de Janeiro (UERJ) Rua São Francisco Xavier 524 Rio de Janeiro RJ20550900 Brazil; d Instituto de Ciências Biológicas e da Saúde Universidade Federal Rural do Rio de Janeiro Seropédica RJ23890000 Brazil; e COPPE Universidade Federal do Rio de Janeiro (UFRJ) Centro de Tecnologia, Cidade Universitária Rio de Janeiro RJ21941914 Brazil; ESRF – The European Synchrotron, France

**Keywords:** phase-contrast imaging, herpetology, entomology, zooarchaeology, palaeontology

## Abstract

The high-energy imaging beamline MOGNO was recently designed, installed and commissioned at the Sirius fourth-generation synchrotron radiation source at the LNLS in Brazil. MOGNO, a micro- and nano-imaging beamline, has as primary source a 3.2 T superbend permanent magnet dipole with a critical energy of 19.15 keV and operates in a cone-beam geometry. The present paper addresses the commissioning experiments in propagation-based phase-contrast imaging and microtomography, revealing high-precision details of a wide range of non-stained biological samples with minimal preparation. We illustrate the potential of non-destructive fast high-resolution microtomographic imaging, particularly for fine anatomical studies of Brazilian biological specimens. Three-dimensional investigations reveal the internal morphology of the head of a dengue fever vector mosquito (*Aedes aegypti*) and of the whole embryo of a reptile (*Brasiliscincus agilis*) and of an amphibian (*Eleutherodactylus cochranae*), as well as the features inherent in an archaeological artefact (*Galeocerdo cuvier* tooth) and a fish fossil bone (Elopomorpha *incertae sedis*).

## Abstract 2

From whole specimens to histological level reconstructions, images derived from 3D synchrotron-based microtomography (SR-microCT) have emerged as a tool that is revolutionizing a wide range of biological research. This paper presents the first commissioning results from experiments to image the anatomy of small animals and specimens from zooarchaeology and palaeontology carried out on MOGNO, the nano- and microtomography beamline at Sirius/LNLS, Brazil.

## 1. Introduction

X-ray synchrotron imaging techniques have been applied to address research problems in different fields, including biomedicine, engineering, materials science, environmental science, geology, archaeology, palaeontology, zoology, food and agriculture (Abrami *et al.*, 2005 ▸; Tafforeau *et al.*, 2006 ▸; Beckmann *et al.*, 2007 ▸; Betz *et al.*, 2007 ▸; Zehbe *et al.*, 2010 ▸; Fusseis *et al.*, 2014 ▸; Broeckhoven & du Plessis, 2018 ▸; Rawson *et al.*, 2020 ▸; Indore *et al.*, 2022 ▸). High-resolution imaging of soft tissues in the sub-micrometre range has undergone improvements, largely due to exploiting the high brilliance, high grade of coherence, high photon flux and small angular beam divergence of synchrotron sources and advanced detector technologies. Biomedical imaging of soft tissue is aided particularly by the faster acquisition time at these sources, which is proportional to the brightness, and the consequent improved spatial resolution, with minimal sample preparation required to produce 3D images with higher signal-to-noise ratio (Suortti & Thomlinson, 2003 ▸; Bilderback *et al.*, 2005 ▸; Baruchel *et al.*, 2006 ▸; Sena *et al.*, 2022 ▸; Albers *et al.*, 2024 ▸).

Virtual histology using 3D synchrotron-based microtomography (SR-microCT) has emerged as a nondestructive imaging tool that is revolutionizing a wide range of biological research. It provides a resolution comparable to that of traditional optical microscopy, while offering 3D data that can be virtually sectioned at any thickness and orientation, delivering significantly more information than conventional irreversible thin sections (Saccomano *et al.*, 2018 ▸; Albers *et al.*, 2018 ▸; Sena *et al.*, 2022 ▸; Mizutani & Suzuki, 2012 ▸). Over the past 30 or so years, synchrotron X-ray phase-contrast imaging (SR-PCI) has been shown to be ideal for visualizing weakly absorbing tissues, based on the intrinsic phase shift which X-rays undergo as they traverse the imaged object (Gureyev *et al.*, 2014 ▸; Croton *et al.*, 2018 ▸; Fidalgo *et al.*, 2020 ▸; Norvik *et al.*, 2020 ▸; Donato & Bonazza, 2024 ▸). The simplest X-ray phase-contrast method, known as propagation-based imaging (PBI), makes use of the Fresnel diffraction of X-rays to yield enhanced visibility of the edges and boundaries within a sample. In PBI mode, the wavefront propagates in an appropriate drift-free space from the source to the sample. The wavefront disturbed by the sample then leads to edge enhancement effects, and phase shifting properties of the probed sample can be visualized in the imaging detector on a microscopic level (Snigirev *et al.*, 1995 ▸; Cloetens *et al.*, 1996 ▸; Momose, 2017 ▸; Endrizzi, 2018 ▸; Tao *et al.*, 2021 ▸). Several reviews have highlighted the unique capabilities of SR-PCI as a valuable tool for a wide range of applications related to life sciences and biomedical research on soft and mineralized tissues (Westneat *et al.*, 2008 ▸; Zhou & Brahme, 2008 ▸; Bravin *et al.*, 2013 ▸; Wilkins *et al.*, 2014 ▸; Quenot *et al.*, 2022 ▸; Claro *et al.*, 2023 ▸).

Fourth-generation synchrotron sources, such as Sirius, the new 3.0 GeV Brazilian Synchrotron Light Source (Liu *et al.*, 2014 ▸; Westfahl *et al.*, 2018 ▸; Craievich, 2020 ▸; Shin, 2021 ▸), provide a fascinating new window of opportunity for bioimaging techniques. Here, we present the first scientific commissioning results from experiments focusing on the anatomy of small animals, zooarchaeology and palaeontology carried out on MOGNO, the nano- and microtomography beamline at Sirius (Archilha *et al.*, 2022 ▸). The aim of this article is to highlight scientific applications of microtomography to reveal high-precision details in the internal morphology of an insect head (*Aedes aegypti* mosquito) and the whole embryo of a reptile (*Brasil­iscincus agilis*) and of an amphibian (*Eleutherodactylus cochranae*), as well as the features inherent in an archaeological artefact (*Galeocerdo cuvier* tooth) and the fossilized bone of a fish (Elopomorpha *incertae sedis*). We illustrate the potential of non-destructive fast high-resolution phase-contrast microtomographic imaging, particularly for fine anatomical studies of Brazilian bio-based specimens.

## 2. Scientific commissioning experiments: samples and setup

### 2.1. MOGNO beamline nanostation

The MOGNO beamline is a high-energy micro- and nanotomography experimental station of Sirius at the Brazilian Synchrotron Light Laboratory (LNLS), a fourth-generation storage ring currently operating at 3 GeV electron energy and 200 mA current. The MOGNO beamline uses a 3.2 T permanent bending magnet as a source. The optical system is optimized for high flux at high photon energy, with a narrow bandwidth (Δ*E*/*E* ≃ 0.13 to 0.01, depending on the energy), while operating in a cone-beam geometry. After the front end, the first optical element is a Pt-coated horizontally focusing elliptical mirror that collects the radiation from the bending magnet source, collimates the beam and creates a secondary source to the subsequent optical system. The second optical system consists of a Kirkpatrick–Baez (KB) mirror system coated with two multilayers, which selects the working energies and focuses the beam to a nanometric spot designed to reach about 120 nm × 120 nm; one of its main advantages is that the X-ray source does not limit the image resolution within the operational range of the beamline. MOGNO is one of the few full-field X-ray tomography beamlines worldwide that operates with focusing optics (KB mirrors). The first stripe provides two different energies, 22 keV and 39 keV (first and second harmonics), and the second stripe provides only 67 keV, which significantly reduces the radiation dose – particularly important for biological samples (Moraes *et al.*, 2023 ▸; Hamann *et al.*, 2025 ▸). In addition, the long source-to-detector distance allows for a wide range of Fresnel numbers, from fractions up to hundreds, enabling experiments in both absorption and Fresnel imaging regimes. A detailed description of the instrumentation and geometric parameters can be found in Archilha *et al.* (2022 ▸).

The MOGNO beamline has two end-stations. Imaging experiments can be carried out using either the microtomography station or the nanostation. MOGNO is a beamline where a cone-beam geometry is implemented, making it possible to perform experiments with pixel sizes from 200 nm with a beam size at the sample of 250 µm in the nanostation. Herein we report on the main features of the nanotomography station. This station holds a ∼7 m granite rail, the sample stage and the detector gantry that only serves the nanostation. The sample stage is based on a set of wedge-shaped granite tables, designed to have three degrees of freedom (*X*, *Y* and *Z*), and all the movements are based on air bearings (Archilha *et al.*, 2022 ▸).

**Figure 1:**
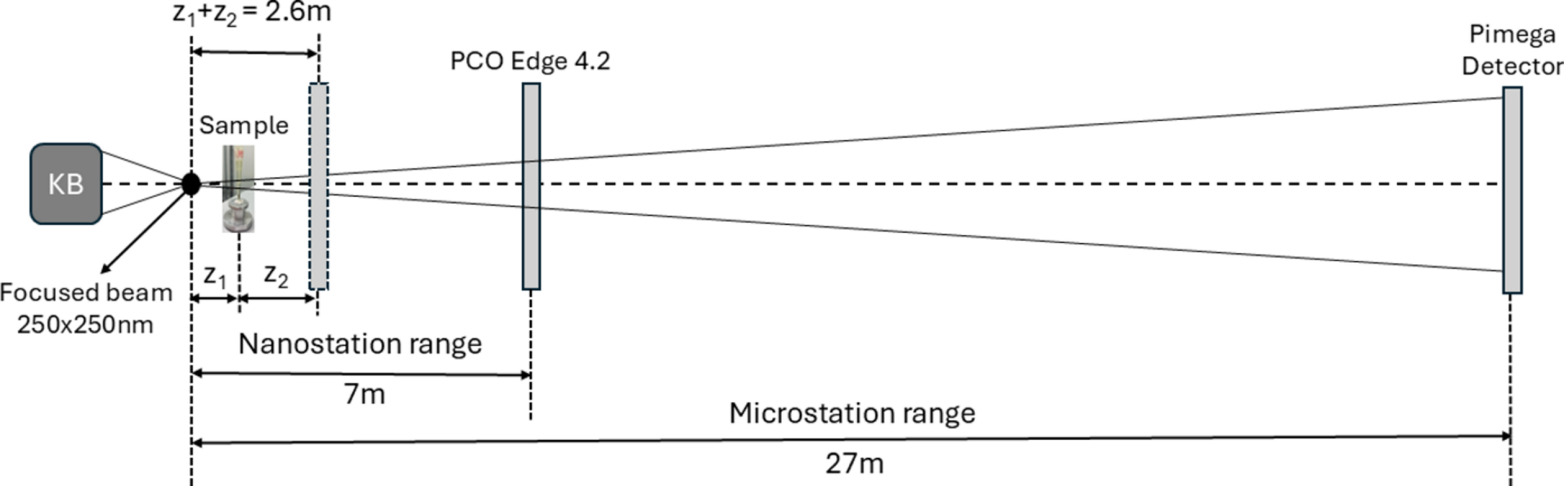
Schematic diagram and positioning of the sample for exposure on the MOGNO beamline. The KB system consists of two multilayer mirrors that focus the X-ray beam in both directions. Note that there is an overlap between the two experimental stations, which covers FoVs between 3.0 and 18.4 mm.

The divergent X-ray beam generated by nanoscale focusing allows selectable magnification, resolution and field of view by changing the source-to-sample distance *z*_1_. The sample can move along the *Z* axis from the KB system’s focus up to the detector. For zoom tomography measurements, we simply adjust the effective pixel size by reducing the source-to-sample distance, positioning the sample along the *Z* axis to achieve the desired zoom level (Archilha *et al.*, 2024 ▸). In the *Experimental results* section[Sec sec3] we provide a detailed analysis of the morphology of an *Aedes aegypti* mosquito head, highlighting the effectiveness of zoom tomography. The experimental setup is sketched in Fig. 1 ▸.

During the present experiment, the focus size was approximately 250 nm × 250 nm, the chosen energy was quasi-monochromatic radiation of 22 keV (Δ*E*/*E* ≃ 0.129) and the exposure time was 1.5 s per projection. We used a PCO edge 4.2 sCMOS (scientific complementary metal–oxide–semiconductor) camera, 2048 × 2048 pixels, with a physical pixel size of 6.5 µm × 6.5 µm. The detector is coupled with an optical magnification system (Optique Peter, Lentilly, France) which consists of a scintillating screen (100 µm LuAG:Ce, Crytur) and 2× or 5× magnifying objective lenses.

This study involved careful optimization of experimental parameters related to the effective pixel size and field of view for various biological samples. All samples were scanned using the detector positioned at 2.6 m (*z*_1_ + *z*_2_) from the source with a 2× objective lens (pixel size *p* = 3.61 µm × 3.61 µm, corresponding to a field of view FoV = 7.40 mm × 7.40 mm). The possibility of varying *z*_1_ allows the FoV, and hence the pixel size, to be adjusted in a flexible manner. The FoV was calculated by FoV = effective pixel size (σ_eff_) × number of detector pixels. The geometric magnification *m* = (*z*_1_ + *z*_2_)/*z*_1_ resulted in an effective propagation distance *z*_eff_ = *z*_2_/*m* and an effective pixel size σ_eff_ = *p*/*m* (Bartels *et al.*, 2013 ▸; Fuhse *et al.*, 2006 ▸). The number of projections was chosen based on the diameter of the volume to be covered by the scan. Phase contrast images were obtained at 1024 and 2048 projections distributed over 180° and 360°, respectively.

The propagation can be fully described by a single parameter, the Fresnel number *F* = *a*^2^/λ*z*_2_, with wavelength λ, sample-to-detector distance *z*_2_ and a typical feature size *a*. For *F* ≃ 1, diffraction effects are significant and images are in the direct-contrast regime, showing pronounced edge enhancement, which can be described *e.g.* by the transport of intensity equation (TIE) (Paganin *et al.*, 2002 ▸). Here, we defined a typical feature size of ten pixels (*a* = 10σ_eff_) and the corresponding discrete Fresnel number *F*^10^ in the case of effective variables in the cone-beam geometry *F*^10^ = 100(σ_eff_)^2^/λ*z*_eff_ (Krenkel *et al.*, 2015 ▸, 2016 ▸; Töpperwien *et al.*, 2018 ▸; Brombal, 2020 ▸); Levine *et al.*, 2021 ▸). Therefore, at *F*^10^ ≃ 10 the object is well recognized. One often speaks of direct contrast images acquired in the near-field propagation regime, which corresponds to large Fresnel numbers *F*^10^ > 10. On the other hand, Fresnel numbers *F*^10^ ≃ 1 and *F*^10^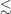 0.1 correspond to the near and far holographic regimes, respectively.

Acquisition parameters such as scan range, number of projections, source-to-detector and sample-to-detector distances, the corresponding FoV, effective pixel sizes and discrete Fresnel numbers calculated for each sample are listed in Table 1 ▸.

In a cone-beam system like MOGNO’s, the resulting geometric resolution σ_R_ is a function of the source size σ_s_, the pixel size of the detector σ_D_ and the local magnification *m*, which in turn depends on the source-to-sample (*z*_1_) and sample-to-detector (*z*_2_) distances (Table 1 ▸), according to the following relation (Bartels *et al.*, 2013 ▸; Krenkel *et al.*, 2015 ▸),
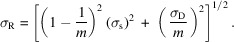
During the experiments, the beamline source size was approximately 250 nm × 250 nm; therefore, in most cases, the geometric resolution was not limited by the source size.

**Figure 2:**
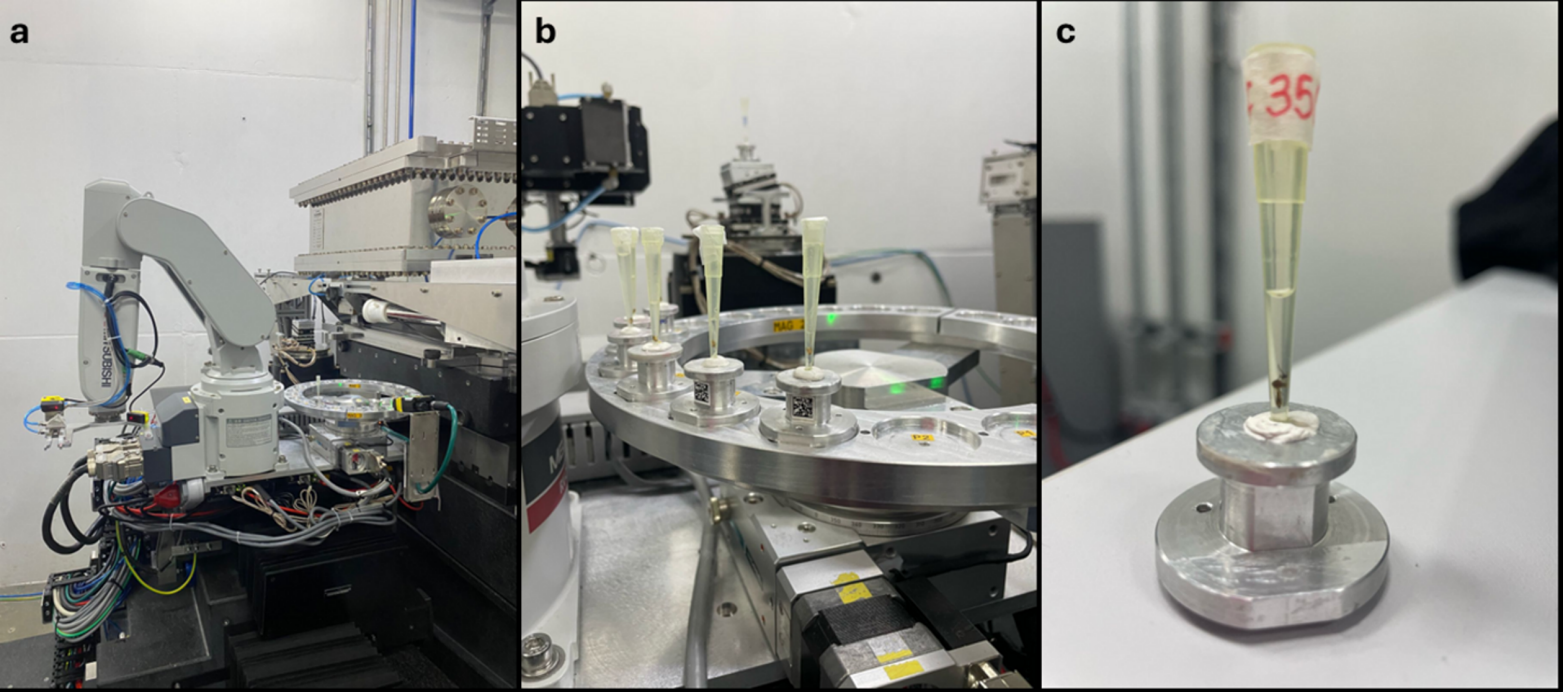
(*a*) View of the experimental setup of the MOGNO nanostation, showing the robotic arm and rotational stage. (*b*) Sample tray. (*c*) Sample holder showing, as an example, a mosquito sample properly adjusted at the bottom of the pipette tip.

The beamline is equipped with an automatic sample exchange system, which was used to position and align the samples in front of the beam. This enables fast sample exchange through an easy-to-use graphical user interface. The robot installed is a Mitsubishi RV-2F-D1 (controller CR-750D) [Fig. 2 ▸(*a*)]. The imaging group designed a sample tray with 22 positions [Fig. 2 ▸(*b*)] and a new sample holder system [Fig. 2 ▸(*c*)] for the beamline. The sample holder has a conical base, which facilitates handling and fitting it on the rotational stage by the robot. Two magnets are responsible for applying a small force, helping to correct the fit and keep it in contact with the base. The robot was programmed to place and remove the sample holder from the rotational stage, where the sample holder is placed for the measurement (Costa *et al.*, 2017 ▸).

Soft-tissue samples (mosquito and embryos) were transferred to polypropyl­ene pipette tips filled with 100% propan-2-ol (absolute alcohol) placed on the top of the sample holder [Fig. 2 ▸(*c*)]. The mounting pipette tips were sealed with parafilm to avoid evaporation during the scan. Both of the valuable and irreplaceable archaeological and paleontological specimens were scanned in empty pipette tips.

Raw data images were corrected for dark current, flat fields and ring artefacts. Subsequently, a single-distance phase-retrieval algorithm, based on the homogeneous TIE (Paganin *et al.*, 2002 ▸), was applied. The slices were then reconstructed with an open-source software tool (Brun *et al.*, 2017 ▸) used to perform phase retrieval (Paganin’s algorithm) and reconstructions via a GPU-based filter back projection (FBP) with Shepp–Logan filtering. Therefore, in this study, the ‘standard’ reconstruction workflow was used, *i.e.* flat fielding, Paganin’s phase retrieval and FBP. Avizo Fire 8 was utilized for the image processing of the datasets, segmentation and morphological analysis of the 3D volumes.

Hereinafter, the terms PCI and SR-microCT are used as synonyms throughout the manuscript to refer to propagation-based phase-contrast imaging.

### 2.2. Sample descriptions

#### 2.2.1. *Aedes aegypti* mosquito

Morphological investigations of disease-transmitting insects still play a key role in the intersections between biology and medicine. Whether in elucidating drug action mechanisms (Erler *et al.*, 2024 ▸) or analysing the transmission of human pathologies (Prüßing *et al.*, 2013 ▸; Sena *et al.*, 2015 ▸), these insects constitute an important subject of study. In this work, we focus on *Aedes aegypti*, a mosquito of significant sanitary importance due to its role in transmitting diseases such as dengue, chikungunya, yellow fever and Zika (Souza-Neto *et al.*, 2019 ▸). Despite its notoriety, detailed morphological studies of *A. aegypti* remain limited to predominantly destructive techniques, such as histological sections, or staining methods that require a chemical agent to image the specimen (Clark *et al.*, 2005 ▸; Lima *et al.*, 2023 ▸). Visualizing the specimens at the level of the whole organism allows morphological studies to be conducted with the aim of achieving effective vector control, as well as analysing potential morphological alterations caused by insecticidal agents (Soonwera *et al.*, 2022 ▸; Serdeiro *et al.*, 2023 ▸).

The adult *A. aegypti* strain Rockefeller used in this study was obtained through collaboration with the Insect Physiology Laboratory at the Universidade Federal Fluminense (Rio de Janeiro, Brazil). Eggs from a previous breeding were placed in approximately 40 ml of rearing water to hatch. The hatched larvae, after being counted, were maintained in a plastic dish containing dechlorinated water and fed 1 mg of powdered fish food flakes (Tetramin Tropical Flakes) in a 1:1:1 ratio. After collection, *A. aegypti* was fixed in Bouin’s solution for four hours and stored in 70% ethanol.

#### 2.2.2. Embryos of *Eleutherodactylus cochranae* and *Brasiliscincus agilis*

In addition to the more usual interest in studying embryos, such as organogenesis and tissue remodelling (and their application), the interest in this type of sample encompasses other subjects, such as physiology, ecology and evolutionary developmental biology (Evo-Devo). To show the utility of SR-microCT in the study of the anatomy of small animals, being able to observe internal organs nondestructively, we have taken two examples of embryos that ‘contradict’ the widespread developmental logic of amphibians and reptiles. The first one is an embryo of the direct-developing frog *Eleuthero­dactylus cochranae*, a species without the free larval stage (tadpole) characteristic of most known toads and frogs (Callery *et al.*, 2001 ▸). The second one is an embryo of the viviparous skink *Brasiliscincus agilis*, a lizard species that retains the eggs and developing embryos within the female body until development is complete, rather than laying a hard-shell egg (Rocha & Vrcibradic, 1999 ▸).

Both specimens are part of the Herpetological Collection of Universidade Federal Rural do Rio de Janeiro (Rio de Janeiro, Brazil) and were previously stored in formalin 10%. Before the analysis, the specimens were washed in distilled water and then submerged in progressive 10 min alcohol baths, until the absolute, following Fidalgo *et al.* (2020 ▸). These baths are designed to ensure removal of water from the formaldehyde storage and slowly replacing it with alcohol. This prevents the formation of micro-bubbles when water is present in the specimen.

#### 2.2.3. Archaeological and palaeontological specimens

SR-microCT can retrieve noteworthy information from archaeological and palaeontological specimens at high spatial resolution without damaging them (Davesne *et al.*, 2020 ▸; Cupello *et al.*, 2022 ▸; Pansani *et al.*, 2023 ▸). Its non-destructive nature is especially important when dealing with exceptionally rare and valuable materials, such as those examined in the present study.

During the commissioning of the MOGNO beamline, we tested archaeological artefacts from the Camboinhas Sambaqui (Rio de Janeiro, Brazil) to analyse the internal microstructure of a shark tooth modified into an artefact, without damaging this very rare archaeological piece. The *Galeocerdo cuvier* tooth was collected during excavations at the Camboinhas Sambaqui in the years 2022 and 2023. It was assigned to the Museu de Arqueologia de Itaipu (Rio de Janeiro, Brazil) for safekeeping, under the reference number CAM-0199. The specimen was imaged while supported in a plastic pipette.

The palaeontological specimen scanned is a fossil fish of Elopomorpha *incertae sedis*, from the Romualdo Member Fossil Lagerstätte of the Santana Formation, Araripe Basin, Lower Cretaceous of Northeastern Brazil (approximately 110 million years old). The specimen is permanently housed at the Universidade do Estado do Rio de Janeiro (Rio de Janeiro, Brazil) under collection ID UERJ-PMB 168. The volume of osteocyte lacunae, measured using SR-microCT, has previously been successfully used to estimate genome sizes in extinct species (Davesne *et al.*, 2020 ▸, Davesne *et al.*, 2021 ▸). In this study, we scanned a fossilized fin ray, supported in a plastic pipette, to test the feasibility of measuring osteocyte lacunae volume in this fossil specimen.

## 3. Experimental results and discussion

### 3.1. Investigation of insect head morphology

A precise and accurate description of the external and internal structures of insects is fundamental for an expanded understanding of their morphology. This knowledge is essential for comprehending insect physiology and behaviour, with applications in the conservation of ecologically critical species (New, 2009 ▸) and the population control of pathogenic species (Finkler, 2012 ▸). The primary technique for obtaining this detailed information has been animal dissection combined with optical microscopy (Porto *et al.*, 2016 ▸). Nevertheless, this method is destructive, preventing the study of structures in their original spatial arrangement and often leading to distortions during the process (Alba-Alejandre *et al.*, 2019 ▸).

Arthropods, being small animals, present challenges in accessing their micrometric structures. Conventional microCT has been used as a non-destructive alternative for morpho­logical studies in various insect species (Alba-Tercedor *et al.*, 2021 ▸; Ferreira *et al.*, 2024 ▸). However, microCT performed in synchrotron laboratories represents considerable advancement, thanks to its intrinsic characteristics of high brilliance and spatial and temporal coherence.

**Figure 3:**
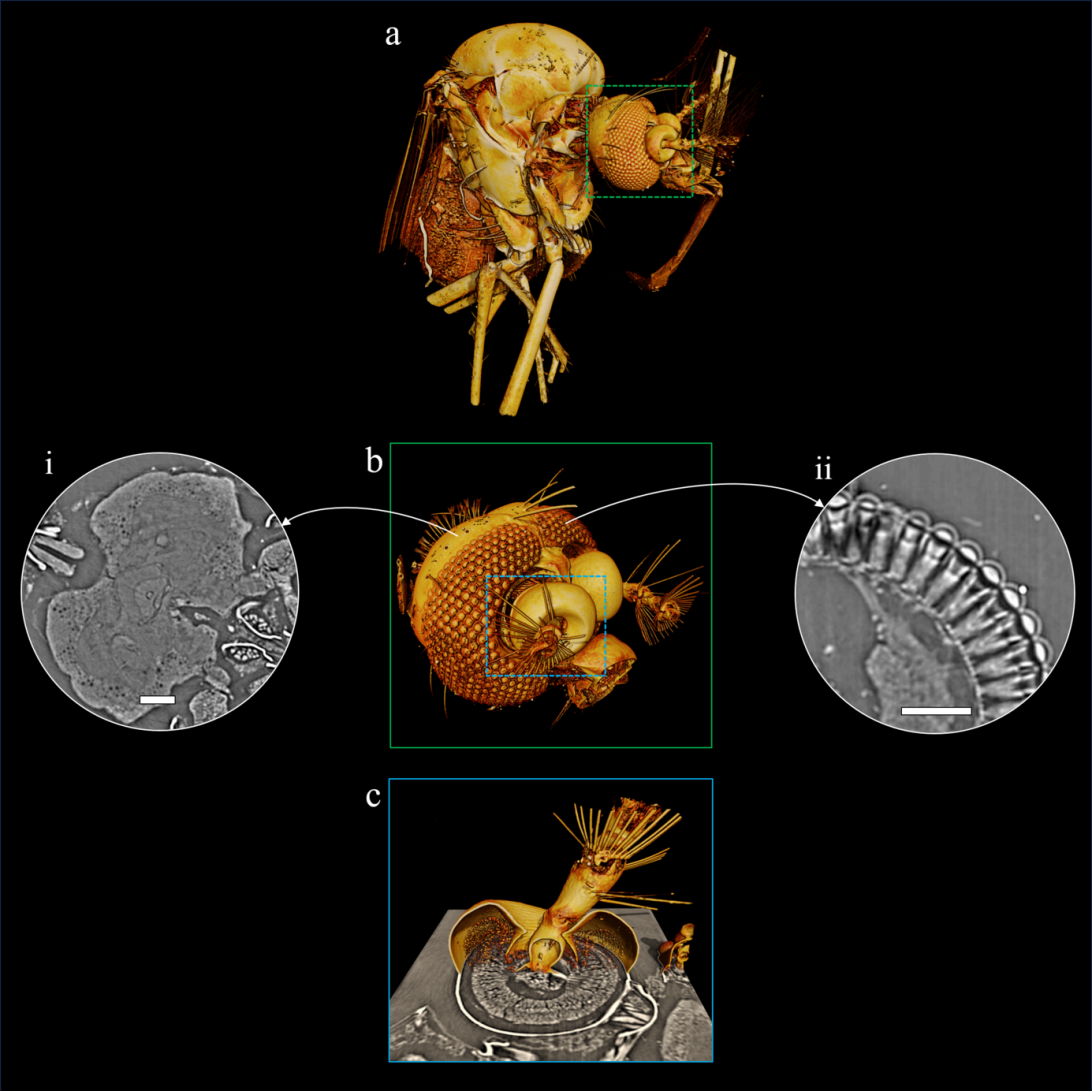
SR-microCT images of *A. aegypti* using different acquisition setups. (*a*) A 3D rendering of the specimen fully scanned with σ_eff_ = 1250 nm. (*b*) The head (green square) acquired with σ_eff_ = 420 nm, highlighting the following internal structures: (i) brain and (ii) compound eye (scale bar 50 µm). (*c*) A 3D rendering and cross section of an antenna (blue square) with σ_eff_ = 280 nm during image acquisition.

SR-microCT has been successfully applied to study the eyes of various butterfly species (Paukner *et al.*, 2024 ▸) and Coleoptera species (Giglio *et al.*, 2022 ▸), the internal organs of beetles (Vommaro *et al.*, 2022 ▸), the internal structures of the bedbug’s head (Sena *et al.*, 2015 ▸), the venation of cricket fossils (Ferreira *et al.*, 2024 ▸) and others. In our study, SR-microCT enabled *in situ* visualization of several biologically relevant subregions of *A. aegypti* through the 3D rendering of the mosquito. Fig. 3 ▸(*a*) allows us to highlight the application of SR-microCT for external morphological study, wherein we can identify legs, wings, palps and antennae, and the spatial distribution of the eyes.

Neurobiological studies have facilitated the exploration and understanding of cognitive functions across diverse insect species. While brain isolation protocols involving dissection exist (Toh *et al.*, 2021 ▸; Wu & Luo, 2006 ▸), this procedure requires high precision due to the extremely small size of the organ. Higher resolution datasets recorded in zoom mode with effective pixel sizes σ_eff_ = 420 nm and σ_eff_ = 280 nm correspond to Fresnel numbers *F*^10^ ≃ 1.2 and *F*^10^ ≃ 0.74, respectively. Due to the smaller Fresnel number *F*^10^ (Table 1 ▸), phase-contrast effects can be observed especially for fine structures in the zoom setting. We successfully visualized the detailed substructures of the *A. aegypti* brain [Fig. 3 ▸(*b*)-i], clearly identifying the nodulus, ellipsoid body, fan-shaped body and mushroom bodies. Beyond the intricate details of the *A. aegypti* brain, Fig. 3 ▸(*b*)-ii offers visualization of the mosquito’s compound eyes, revealing ommatidium structures, the curvature of the cornea, the crystalline cone and axons. These fundamental features align with the early morphological description of the adult *A. aegypti* eye documented by Brammer (1970 ▸), which also detailed the basic arrangement of these optical elements.

In mosquitoes, the olfactory system comprises the mushroom body, antennae and maxillary palps (two of the three sensory appendages/organs) (Konopka *et al.*, 2021 ▸; Bohbot *et al.*, 2013 ▸). Fig. 3 ▸(*c*) demonstrates that SR-microCT enables a dual approach to studying the antenna: visualizing its external structure and conducting histological evaluation. This detailed understanding of the olfactory system in disease-vectoring mosquitoes can significantly contribute to the development of effective disease control strategies, as this system governs their host-seeking behaviour (Vinauger *et al.*, 2014 ▸).

Although SR-microCT is a well established technique in insect studies, no previous research was found applying this method specifically to *A. aegypti*; existing studies have relied solely on conventional microCT (Lima *et al.*, 2023 ▸). The findings presented here therefore demonstrate the novelty and potential of this approach, showcasing the high-resolution imaging and fine morphological detail that can be achieved without the need for chemical staining. These results establish a valuable foundation for future investigations into this pathogenically significant species.

### 3.2. Herpetological specimens

The introduction of microCT techniques to fields of vertebrate zoology, in particular herpetology (the study of amphibians and reptiles), was groundbreaking. It allowed new studies of old problems and even new opportunities for research in morphology, ecology and evolutionary biology (Metscher, 2009 ▸; Faulwetter *et al.*, 2013 ▸; Broeckhoven & du Plessis, 2018 ▸). The different tissues and organs may yield a wide variety of information on different aspects of these animals’ morphology, and in this sense and less explored are ultrastructural and developmental analyses. Ultrastructural analysis is concerned with the subunits of the tissues, their fine structure, differences between regions of morphology and their relationship with each other, whereas developmental analysis is interested in changes in morphology during development.

Among the most commonly studied morphological systems, those associated with the skeleton are essential in vertebrate comparative research, such as developmental biology, morphology, palaeontology and systematics. Studies of living organisms classically involve techniques based on dry specimen preparation or chemical baths, through clearing and staining methods [*e.g.* Dingerkus & Uhler (1977 ▸)]. These techniques render bones red or purple and cartilage blue. However, during the process of preparation all the other systems are lost. Because of their structure, bones have been the most studied system in herpetological specimens using conventional microCT (Broeckhoven & du Plessis, 2018 ▸). In most of these studies, skeletons are described and compared between species [*e.g.* Batista *et al.* (2025 ▸)]. Although SR-microCT has been used in descriptive research involving herpetofauna [*e.g.* Boistel *et al.* (2013 ▸) and Haas *et al.* (2014 ▸)], developmental studies aiming to follow the ontogeny of different systems throughout the developmental stages were carried out only recently (Fidalgo *et al.*, 2018 ▸, 2020 ▸).

In direct-developing frog species, as well as in reptiles, osteogenesis has its onset while still inside the egg (Vera Candioti *et al.*, 2020 ▸; Ollonen *et al.*, 2018 ▸), with each bone (or pair) and species showing its own ossification time and sequence. As with conventional microCT, mineralized structures like bones are easier to observe, therefore we had similar results measuring the state of ossification in both specimens. We were able to identify different bones in early stages of development of the cranial, axial and appendicular elements [Figs. 5(*d*) and 6(*c*)], and we were also able to identify early stages of ossification (Figs. 5 and 6).

**Figure 4:**
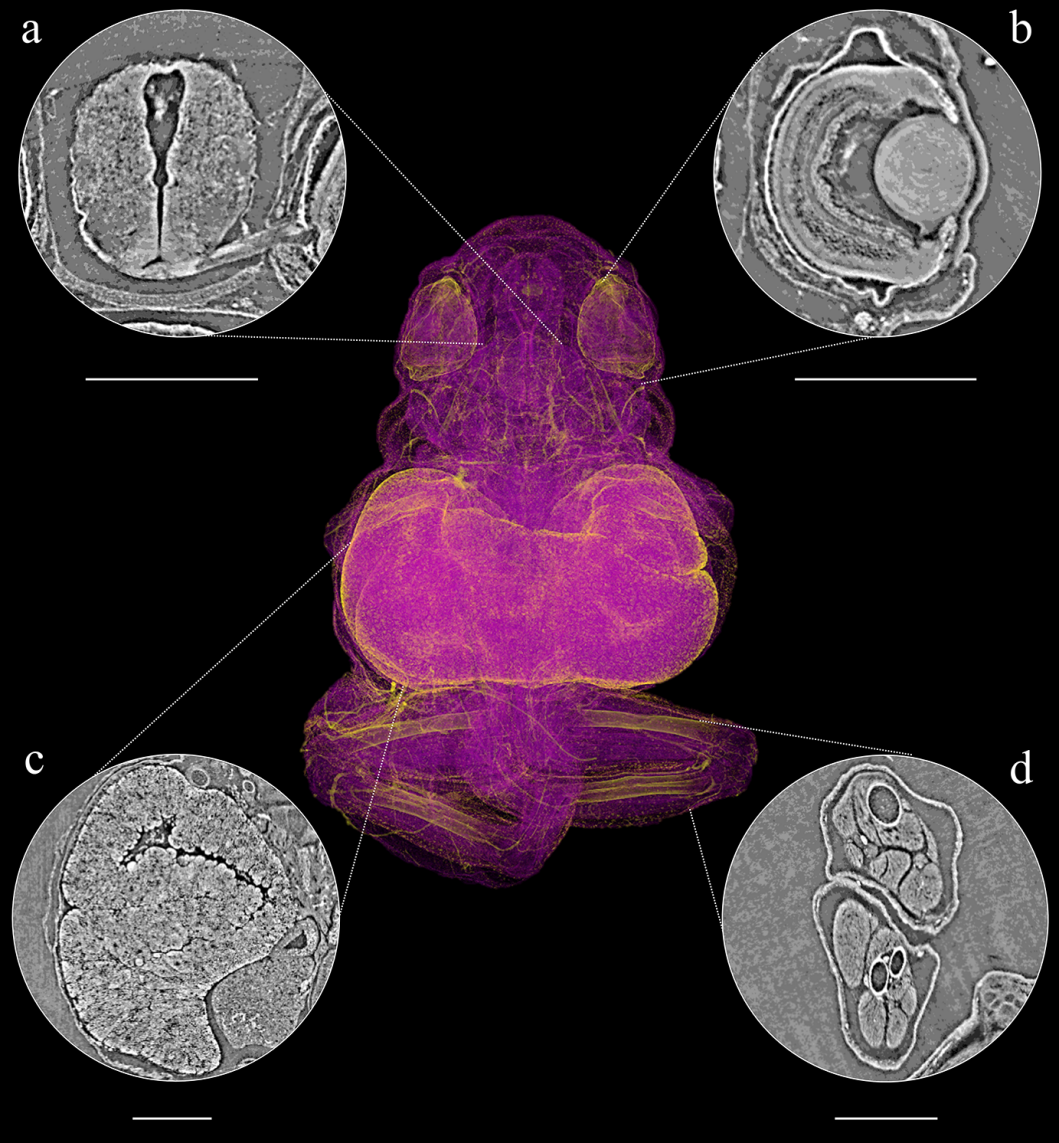
(Centre) Volume rendering of *Eleutherodactylus cochranae* embryo. Around the central image are 2D slices of observed tissues: (*a*) brain, (*b*) eye, (*c*) digestive tract and yolk, and (*d*) leg bones (femur, tibia and fibula) and muscles (scale bar 500 µm).

**Figure 5:**
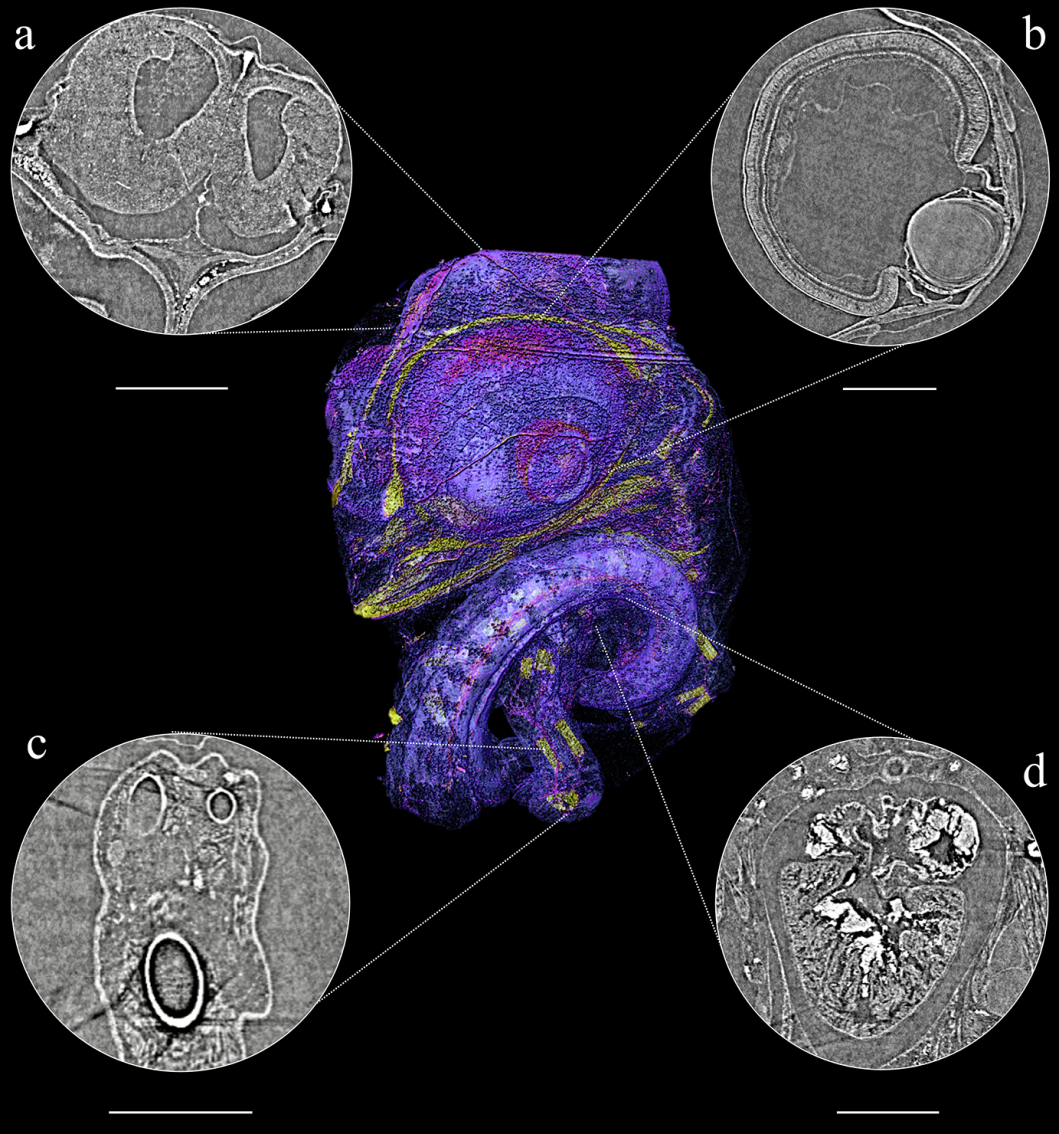
(Centre) Volume rendering of *Brasiliscincus agilis* embryo. Around the central image are 2D slices of observed tissues: (*a*) brain, (*b*) eye, (*c*) leg bones (femur, tibia and fibula), and (*d*) heart (scale bar 500 µm).

In conventional microCT, it is possible to study soft tissue with the use of staining that increases the contrast in such tissues (Metscher, 2009 ▸; Gignac & Kley, 2014 ▸). These techniques permit the visualization of components from different systems, such as muscular, nervous and some visceral. However, the use of staining to study rare specimens is not usually allowed by the collection’s curators, because these techniques may, in the long run, damage the specimens (Broeckhoven & du Plessis, 2018 ▸). The MOGNO beamline allowed us to discriminate between soft-tissue organs, as well as their sub-units (Figs. 4 ▸ and 5 ▸), without staining. For both samples (*E. cochranae* and *B. agilis*), in addition to the developing bones, we were able to observe in detail the eye morphology and the central nervous system (CNS) (Figs. 4 ▸ and 5 ▸). In addition, the brain and the whole CNS were easily observed [Figs. 4 ▸(*a*) and 5 ▸(*a*)], allowing us even to identify specific nerves such as the optic nerve [Fig. 4 ▸(*a*)]. On the eyes, we observed key structures, such as the lens, cornea, retina and their layers [Figs. 4 ▸(*b*) and 5 ▸(*b*)]. Other specific organs and tissues were visualized in only one of the specimens. For instance, in *B. agilis* it was possible to identify the heart and its cavities filled with blood [Fig. 5 ▸(*d*)], whereas in *E. cochranae* it was possible to identify structures associated with the developing digestive tract, filled by the yolk at this stage [Fig. 5 ▸(*c*)].

Although the use of conventional microCT in herpeto­logical research has increased in the last 20 years with a variety of applications (Broeckhoven & du Plessis, 2018 ▸), the number of studies applying SR-microCT to study embryos and larvae and their development and organogenesis are still scarce. The development of protocols and methodologies to measure this type of specimen may open doors to the use of other model animals among members of herpetofauna in different types of study and help to reveal more about the biology of these organisms, with applications from morphology and physiology to ecology and evolutionary biology (Burggren & Warburton, 2007 ▸).

### 3.3. Zooarchaeological artefact

Despite being well documented from an archaeological perspective, many questions remain unanswered regarding the zooarchaeological records of Itaipu and Camboinhas, Niterói, Brazil [*e.g.* Kneip (1979 ▸) and Kneip *et al.* (1981 ▸)]. Archaeological materials often include hard tissues with complex structures down to the subcellular level. In such cases, conventional microCT imaging provides little to no contrast due to insufficient X-ray absorption. Phase-contrast microtomography, achievable only at synchrotron facilities, is therefore essential for accessing the internal anatomy and histology of these rare materials on the (sub-)microscale without causing damage.

**Figure 6:**
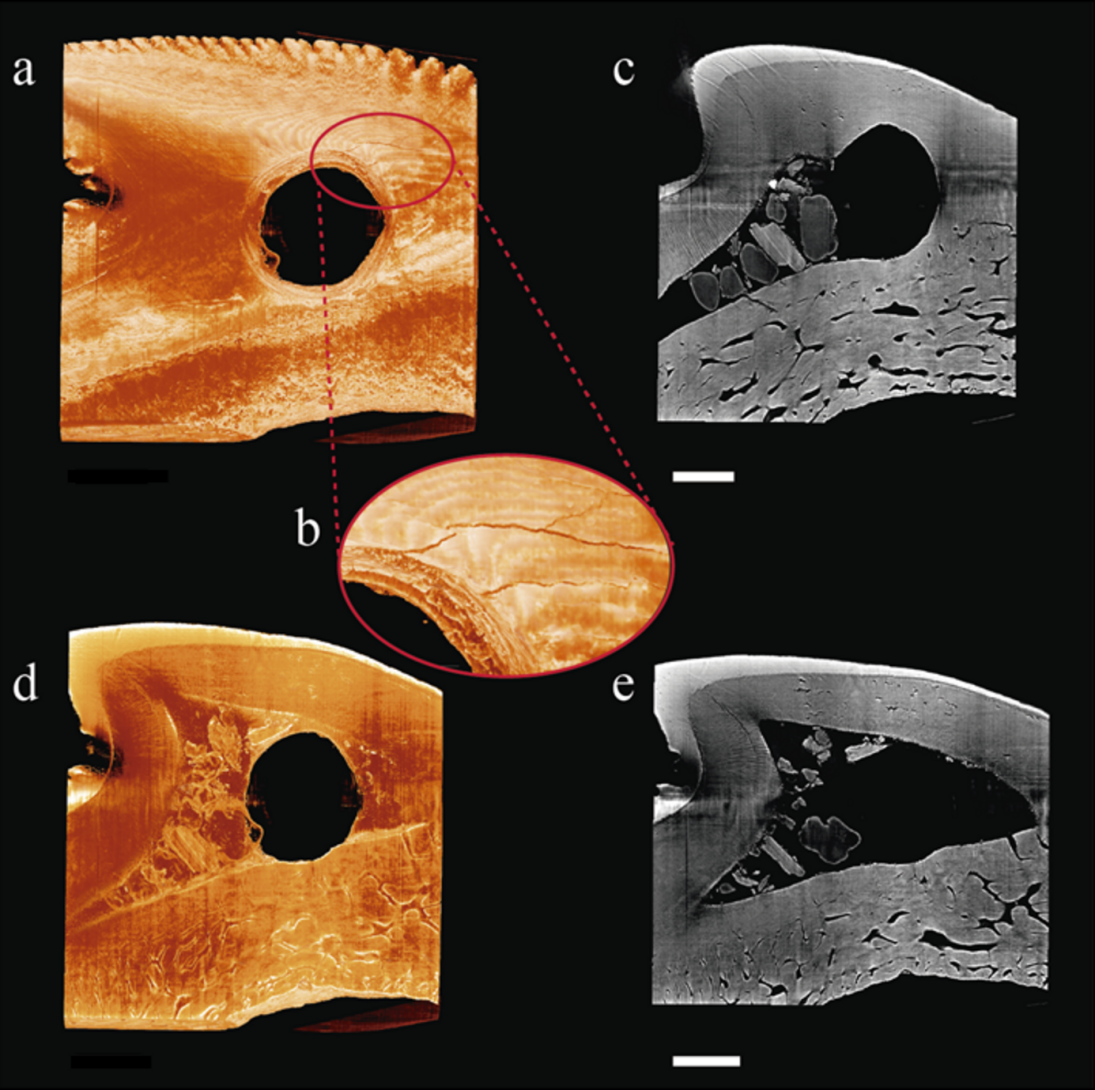
Anthropogenically modified shark tooth CAM-0199 from Camboinhas Sambaqui, Niterói, Rio de Janeiro. (*a*) Three-dimensional reconstruction of the tooth, revealing the central perforation. (*b*) Close-up of the 3D reconstruction in panel (*a*), highlighting cracks inherent to the perforation. (*c*) Coronal section showing the junction between the central perforation and the natural hollow internal structure of the tooth. (*d*) Three-dimensional reconstruction displaying the hollow internal structure. (*e*) Coronal section of the tooth displaying the hollow internal structure. [Scale bars: (*a*), (*c*), (*d*) and (*e*) 1 mm, and (*b*) 0.25 mm.]

The objective of this measurement on the MOGNO beamline was to investigate the natural internal composition of shark teeth to assess whether their anatomy may have facilitated later modifications. The shark tooth analysed in this study (specimen CAM-0199) features a central perforation characteristic of human alteration [Fig. 6 ▸(*a*)]. High-quality images obtained through SR-microCT revealed the hollow internal structure, which probably made drilling easier [Figs. 6 ▸(*c*) and 6 ▸(*e*)], as well as cracks consistent with this modification process [Fig. 6 ▸(*b*)].

The results, based on 3D SR-microCT images, offer a detailed view of the features associated with human-made perforations, highlighting modified internal structures, additional incisions, anthropogenic marks and surface scars [for further examples of this potentiality, see Pansani *et al.* (2023 ▸)].

### 3.4. Osteocyte volume in a palaeontological specimen

Whole-genome duplication (WGD) has frequently been suggested as a key driver behind the remarkable evolutionary diversification of the teleosts (ray-finned bony fishes) (Davesne *et al.*, 2021 ▸). Nevertheless, the lack of genetic information from fossil species has made it difficult to determine the exact timing of the WGD event within teleost evolution (Davesne *et al.*, 2021 ▸). Osteocyte volume in fossil bones is potentially relevant to estimate large-scale changes in genome size in vertebrate evolutionary history (Davesne *et al.*, 2020 ▸). The volume variation of osteocytes (the main cellular component of the mineralized matrix in vertebrate bones) has been shown to correlate with genome size in tetrapods and, more recently, in ray-finned fish species (Hall, 2015 ▸; Davesne *et al.*, 2020 ▸, 2021 ▸).

Traditional methods, such as ground and thin sections, are the most commonly used techniques for estimating osteocyte morphology [*e.g.* Francillon-Vieillot *et al.* (1990 ▸), Sire & Meunier (1994 ▸) and Cupello *et al.* (2017 ▸)]. However, while these methods preserve some information on osteocyte shape and volume, many lacunae are partially sectioned, which limits both the characterization and quantification of their 3D structure (D’Emic & Benson, 2013 ▸; Marotti, 1981 ▸; Stein & Prondvai, 2014 ▸; Davesne *et al.*, 2020 ▸).

**Figure 7:**
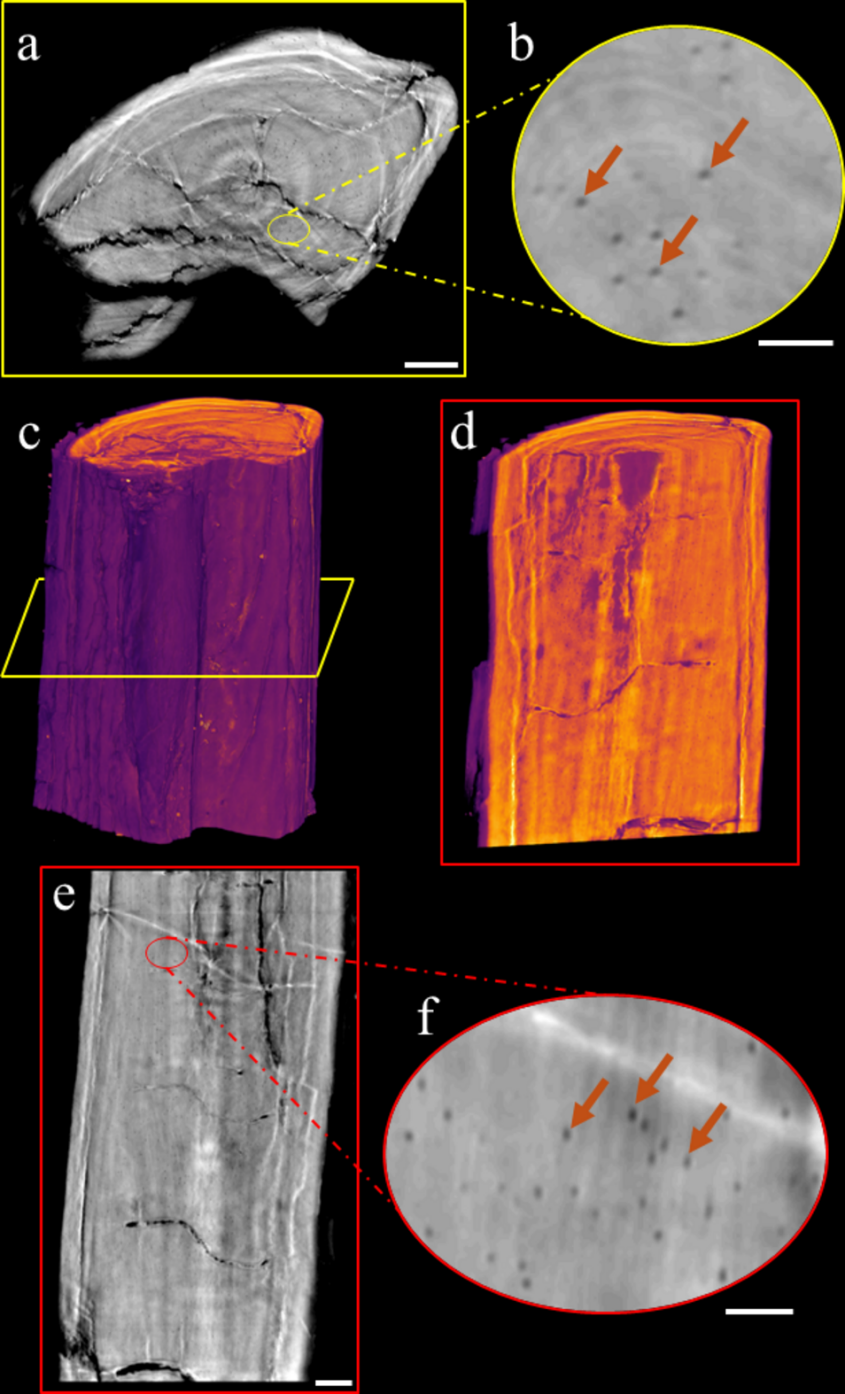
Osteocytes in a fragment of the fossilized fin ray UERJ-PMB 168. (*a*) Transverse section of the fin ray. (*b*) Close-up of the transverse section in panel (*a*), highlighting the osteocytes. (*c*, *d*) Three-dimensional reconstructions of the fin ray. (*e*) Longitudinal section of the fin ray. (*f*) Close-up of the longitudinal section in panel (*e*), highlighting the osteocytes. Orange arrows point to the osteocytes. [Scale bars: (*a*), (*c*), (*d*) and (*e*) 200 µm, and (*b*) and (*f*) 50 µm.]

Synchrotron-based virtual histology is the only non-destructive and effective method for examining bone microstructure in both fossils and extant vertebrates (Sanchez *et al.*, 2012 ▸). Thanks to the propagation phase-contrast microtomography of the MOGNO beamline, the measurement of a fragment of a fossilized fin ray from Elopomorpha *incertae sedis* (sample UERJ-PMB 168) clearly revealed osteocyte lacunae [Figs. 7 ▸(*a*), 7 ▸(*b*), 7 ▸(*e*) and 7 ▸(*f*)] that can be measured and compared with extant taxa.

## 4. Conclusion

This paper presents the promising initial commissioning output from the MOGNO X-ray micro- and nanotomography beamline at the Sirius storage ring, a fourth-generation synchrotron source built at the LNLS, Brazil. We have described the capabilities of the beamline for imaging millimetre-sized biological samples, highlighting the performance and potential of the nanostation to produce high-resolution 3D images.

Case studies of the overall morphology of small vertebrate and invertebrate animals, including delicate and unique specimens from museum collections, are presented. These results demonstrate the ability to perform virtual dissections of samples in any desired plane without compromising their integrity.

These successful scientific outcomes are regarded as a significant advancement towards the potential of the MOGNO beamline being made available to users, which is expected to increase the use of SR-microCT for many applications in biological and biomedical fields.

## Figures and Tables

**Table 1:** Experimental parameters

Sample	Scan range (°)	Number of projections	*z*_1_ (m)	*z*_2_ (m)	FoV (mm)	σ_eff_ (µm)	*F* ^10^
*Aedes aegypti* mosquito	180	1024	0.90	1.70	2.56	1.2	4.7
			0.30	2.30	0.85	0.42†	1.2
			0.20	2.40	0.57	0.28†	0.74
*Eleutherodactylus cochranae* embryo	360	2048	1.83	0.77	5.20	2.5	21
*Brasiliscincus agilis* embryo	180	1024	2.13	0.47	6.06	3.0	40
*Galeocerdo cuvier* tooth	360	2048	2.13	0.47	6.06	3.0	40
Fossilized fin ray	360	2048	1.10	1.50	3.13	1.5	6.5

† Images obtained in zoom mode.

## Data Availability

The data that support the findings of this study are available from the corresponding author, MVC, upon reasonable request.

## References

[bb1] Abrami, A., Arfelli, F., Barroso, R. C., Bergamaschi, A., Billè, F., Bregant, P., Brizzi, F., Casarin, K., Castelli, E., Chenda, V., Dalla Palma, L., Dreossi, D., Fava, C., Longo, R., Mancini, L., Menk, R.-H., Montanari, F., Olivo, A., Pani, S., Pillon, A., Quai, E., Ren Kaiser, S., Rigon, L., Rokvic, T., Tonutti, M., Tromba, G., Vascotto, A., Venanzi, C., Zanconati, F., Zanetti, A. & Zanini, F. (2005). *Nucl. Instrum. Methods Phys. Res. A* **548**, 221–227.

[bb2] Alba-Alejandre, I., Alba-Tercedor, J. & Vega, F. E. (2019). *Sci. Rep.* **9**, 17150. 10.1038/s41598-019-53537-z PMC6868283 31748574

[bb3] Alba-Tercedor, J., Hunter, W. B. & Alba-Alejandre, I. (2021). *Sci. Rep.* **11**, 1358. 10.1038/s41598-020-80404-z PMC7809155 33446699

[bb4] Albers, J., Svetlove, A. & Duke, E. (2024). *J. Cell Sci.* **137**, jcs261953. 10.1242/jcs.261953 PMC11529875 39440473

[bb5] Albers, J., Pacilé, S., Markus, M. A., Wiart, M., Vande Velde, G., Tromba, G. & Dullin, C. (2018). *Mol. Imaging Biol.* **20**, 732–741. 10.1007/s11307-018-1246-3 29968183

[bb6] Archilha, N. L., Costa, G. R., Ferreira, G. R. B., Moreno, G. B. Z. L., Rocha, A. S., Meyer, B. C., Pinto, A. C., Miqueles, E. X. S., Cardoso, M. B. & Westfahl, H. Jr (2022). *J. Phys. Conf. Ser.* **2380**, 012123.

[bb7] Archilha, N. L., Costa, G. S., Oliveira, J. F., Miqueles, E. X. & Ferreira, T. R. (2024). *Synchrotron Rad. News* **37**(5), 34–39.

[bb8] Bartels, M., Hernandez, V. H., Krenkel, M., Moser, T. & Salditt, T. (2013). *Appl. Phys. Lett.* **103**, 083703.

[bb9] Baruchel, J., Buffiere, J. Y., Cloetens, P., Di Michiel, M., Ferrie, E., Ludwig, W., Maire, E. & Salvo, L. (2006). *Scr. Mater.* **55**, 41–46.

[bb10] Batista, M., Limp, G., Colaço, G., de Oliveira, S. C. G., Paiva, K., Colaço, M., Barroso, R. C. & da Silva, H. R. (2025). *Zool. Anz.* **316**, 266–274.

[bb11] Beckmann, F., Grupp, R., Haibel, A., Huppmann, M., Nöthe, M., Pyzalla, A., Reimers, W., Schreyer, A. & Zettler, R. (2007). *Adv. Eng. Mater.* **9**, 939–950.

[bb12] Betz, O., Wegst, U., Weide, D., Heethoff, M., Helfen, L., Lee, W. K. & Cloetens, P. (2007). *J. Microsc.* **227**, 51–71. 10.1111/j.1365-2818.2007.01785.x 17635659

[bb13] Bilderback, D. H., Elleaume, P. & Weckert, E. (2005). *J. Phys. B At. Mol. Opt. Phys.* **38**, S773–S797.

[bb14] Bohbot, J. D., Durand, N. F., Vinyard, B. T. & Dickens, J. C. (2013). *Front. Physiol.* **4**, 39. 10.3389/fphys.2013.00039 PMC3590643 23471139

[bb15] Boistel, R., Aubin, T., Cloetens, P., Peyrin, F., Scotti, T., Herzog, P., Gerlach, J., Pollet, N. & Aubry, J.-F. (2013). *Proc. Natl Acad. Sci. USA* **110**, 15360–15364. 10.1073/pnas.1302218110 PMC3780892 24003145

[bb16] Brammer, J. D. (1970). *J. Exp. Zool.* **175**, 181–195.

[bb17] Bravin, A., Coan, P. & Suortti, P. (2013). *Phys. Med. Biol.* **58**, R1–R35. 10.1088/0031-9155/58/1/R1 23220766

[bb18] Broeckhoven, C. & du Plessis, A. (2018). *Amphib. Reptilia* **39**, 377–401.

[bb19] Brombal, L. (2020). *J. Instrum.* **15**, C01005.

[bb20] Brun, F., Massimi, L., Fratini, M., Dreossi, D., Billé, F., Accardo, A., Pugliese, R. & Cedola, A. (2017). *Adv. Struct. Chem. Imag* **3**, 4. 10.1186/s40679-016-0036-8 PMC5313567 28261542

[bb21] Burggren, W. W. & Warburton, S. (2007). *ILAR J.* **48**, 260–269. 10.1093/ilar.48.3.260 17592189

[bb101] Callery, E. M., Fang, H. & Elinson, R. P. (2001). *BioEssays*, **23**, 233–241. 10.1002/1521-1878(200103)23:3<233::AID-BIES1033>3.0.CO;2-Q 11223880

[bb22] Clark, T. M., Hutchinson, M. J., Huegel, K. L., Moffett, S. B. & Moffett, D. F. (2005). *Tissue Cell* **37**, 457–468. 10.1016/j.tice.2005.08.001 16221479

[bb23] Claro, P. I. C., Borges, E. P. B. S., Schleder, G. R., Archilha, N. L., Pinto, A., Carvalho, M., Driemeier, C. E., Fazzio, A. & Gouveia, R. F. (2023). *Appl. Phys. Rev.* **10**, 021302.

[bb24] Cloetens, P., Barrett, R., Baruchel, J., Guigay, J. P. & Schlenker, M. (1996). *J. Phys. D Appl. Phys.* **29**, 133–146.

[bb25] Costa, G., Archilha, N. L., O’Dowd, F. & Vasconcelos, G. (2018). *Proceedings of the 16th International Conference on Accelerator and Large Experimental Physics Control Systems (ICALEPCS2017)*, Barcelona, Spain, 8–13 October 2017. TUPHA203.

[bb26] Craievich, A. F. (2020). *Radiat. Phys. Chem.* **167**, 108253.

[bb27] Croton, L. C. P., Morgan, K. S., Paganin, D. M., Kerr, L. T., Wallace, M. J., Crossley, K. J., Miller, S. L., Yagi, N., Uesugi, K., Hooper, S. B. & Kitchen, M. J. (2018). *Sci. Rep.* **8**, 11412. 10.1038/s41598-018-29841-5 PMC6065359 30061729

[bb28] Cupello, C., Hirasawa, T., Tatsumi, N., Yabumoto, Y., Gueriau, P., Isogai, S., Matsumoto, R., Saruwatari, T., King, A., Hoshino, M., Uesugi, K., Okabe, M. & Brito, P. M. (2022). *eLife* **11**, e77156. 10.7554/eLife.77156 PMC9323002 35880746

[bb29] Cupello, C., Meunier, F. J., Herbin, M., Janvier, P., Clément, G. & Brito, P. M. (2017). *Sci. Rep.* **7**, 9244. 10.1038/s41598-017-09327-6 PMC5569016 28835617

[bb30] Davesne, D., Friedman, M., Schmitt, A. D., Fernandez, V., Carnevale, G., Ahlberg, P. E., Sánchez, S. & Benson, R. B. J. (2021). *Proc. Natl Acad. Sci. USA* **118**, e2101780118. 10.1073/pnas.2101780118 PMC8325350 34301898

[bb31] Davesne, D., Schmitt, A. D., Fernandez, V., Benson, R. B. & Sanchez, S. (2020). *J. Evol. Biol.* **33**, 808–830. 10.1111/jeb.13612 32144878

[bb32] D’Emic, M. D. & Benson, R. B. J. (2013). *Bone* **57**, 300–310. 10.1016/j.bone.2013.08.010 23954754

[bb33] Dingerkus, G. & Uhler, L. D. (1977). *Stain Technol.* **52**, 229–232. 10.3109/10520297709116780 71769

[bb34] Donato, S. & Bonazza, D. (2024). *Trends Anal. Chem.* **180**, 117943.

[bb35] Endrizzi, M. (2018). *Nucl. Instrum. Methods Phys. Res. A* **878**, 88–98.

[bb36] Erler, S., Cotter, S. C., Freitak, D., Koch, H., Palmer-Young, E. C., de Roode, J. C., Smilanich, A. M. & Lattorff, H. M. G. (2024). *Trends Parasitol.* **40**, 338–349. 10.1016/j.pt.2024.02.003 38443305

[bb37] Faulwetter, S., Vasileiadou, A., Kouratoras, M., Dailianis, T. & Arvanitidis, C. (2013). *ZooKeys* **263**, 1–45. 10.3897/zookeys.263.4261 PMC3591762 23653515

[bb38] Ferreira, J., Josse, H., Denadai de Campos, L., Nel, A. & Desutter-Grandcolas, L. (2024). *Foss. Rec.* **27**, 101–110.

[bb102] Fidalgo, G., Colaço, M. V., Nogueira, L. P., Braz, D., Silva, H. R., Colaço, G. & Barroso, R. C. (2018). *J. Instrum.* **13**, C05012.

[bb39] Fidalgo, G., Paiva, K., Mendes, G., Barcellos, R., Colaço, G., Sena, G., Pickler, A., Mota, C. L., Tromba, G., Nogueira, L. P., Braz, D., Silva, H. R., Colaço, M. V. & Barroso, R. C. (2020). *Sci. Rep.* **10**, 18934. 10.1038/s41598-020-75993-8 PMC7641268 33144603

[bb40] Finkler, C. L. L. (2012). *An. Acad. Pernambucana Cienc. Agron.* **8**, 169–189.

[bb41] Francillon-Vieillot, H., de Buffrénil, V., Castanet, J., Geraudie, J., Meunier, F., Sire, J.-Y., Zylberberg, L. & de Ricqlès, A. (1990). *Skeletal Biomineralization: Patterns, Processes and Evolutionary Trends*, edited by J. G. Carter, pp. 471–548. New York: Van Nostrand Reinhold.

[bb42] Fuhse, C., Ollinger, C. & Salditt, T. (2006). *Phys. Rev. Lett.* **97**, 254801. 10.1103/PhysRevLett.97.254801 17280360

[bb43] Fusseis, F., Xiao, X., Schrank, C. & De Carlo, F. (2014). *J. Struct. Geol.* **65**, 1–16.

[bb44] Giglio, A., Vommaro, M. L., Agostino, R. G., Lo, L. K. & Donato, S. (2022). *Life* **12**, 741. 10.3390/life12050741 PMC9145526 35629408

[bb45] Gignac, P. M. & Kley, N. J. (2014). *J. Exp. Zool. B* **322**, 166–176. 10.1002/jez.b.22561 24482316

[bb46] Gureyev, T. E., Mayo, S. C., Nesterets, Y. I., Mohammadi, S., Lockie, D., Menk, R. H., Arfelli, F., Pavlov, K. M., Kitchen, M. J., Zanconati, F., Dullin, C. & Tromba, G. (2014). *J. Phys. D Appl. Phys.* **47**, 365401.

[bb47] Haas, A., Pohlmeyer, J., McLeod, D. S., Kleinteich, T., Hertwig, S. T., Das, I. & Buchholz, D. R. (2014). *Zoomorphology* **133**, 321–342.

[bb48] Hall, B. K. (2015). *Developmental and Evolutionary Skeletal Biology.* Academic Press.

[bb49] Hamann, J. H., Wozniak, G. S., Ferreira, T. R., Zambianchi, P. & Malthez, A. L. M. C. (2025). *Radiat. Phys. Chem.* **235**, 112864.

[bb50] Indore, N. S., Karunakaran, C. & Jayas, D. S. (2022). *Plant Methods*, **18**, 101. 10.1186/s13007-022-00932-9 PMC9375343 35964094

[bb51] Kneip, L. M. (1979). *Pesquisas de salvamento em Itaipu, Niterói, Rio de Janeiro.* Itaipu Cia de Desenvolvimento Territorial. Rio de Janeiro.

[bb52] Kneip, L. M., Pallestrini, L. & de Souza Cunha, F. L. (1981). *Pesquisas arqueolóicas no litoral de Itaipu, Niterói, Rio de Janeiro.* Ed Gráfica Luna.

[bb53] Konopka, J. K., Task, D., Afify, A., Raji, J., Deibel, K., Maguire, S., Lawrence, R. & Potter, C. J. (2021). *Chem. Senses*, **46**, bjab021. 10.1093/chemse/bjab021 PMC8256107 33885760

[bb54] Krenkel, M., Markus, A., Bartels, M., Dullin, C., Alves, F. & Salditt, T. (2015). *Sci. Rep.* **5**, 9973. 10.1038/srep09973 PMC4428069 25966338

[bb55] Krenkel, M., Töpperwien, M., Dullin, C., Alves, F. & Salditt, T. (2016). *AIP Adv.* **6**, 035007.

[bb56] Levine, Z. H., Garboczi, E. J., Peskin, A. P., Ekman, A. A., Mansfield, E. & Holm, J. D. (2021). *Opt. Express* **29**, 1788. 10.1364/OE.414398 PMC7920526 33726385

[bb57] Lima, M. G., Jussiani, E. I., Andrello, A. C., Zequi, J. A. C. & Kawabata, E. K. (2023). *Micron* **173**, 103518. 10.1016/j.micron.2023.103518 37531794

[bb58] Liu, L., Milas, N., Mukai, A. H. C., Resende, X. R. & de Sá, F. H. (2014). *J. Synchrotron Rad.* **21**, 904–911. 10.1107/S1600577514011928 25177981

[bb59] Marotti, G. (1981). *Bone Histomorphometry*, edited by W. S. S. Jee & A. M. Parfitt, pp. 223–229. Paris: Armour Montagu.

[bb60] Metscher, B. D. (2009). *BMC Physiol.* **9**, 11. 10.1186/1472-6793-9-11 PMC2717911 19545439

[bb61] Mizutani, R. & Suzuki, Y. (2012). *Micron* **43**, 104–115. 10.1016/j.micron.2011.10.002 22036251

[bb62] Momose, A. (2017). *J. Electron Microsc.* **66**, 155–166.

[bb63] Moraes, I. C., Hesterberg, D., Bacchim Neto, F. A., Archilha, N. L., Pérez, C. A., Araújo, M. V. A. & Ferreira, T. R. (2023). *Sci. Rep.* **13**, 5643. 10.1038/s41598-023-32540-5 PMC10079845 37024527

[bb64] New, T. R. (2009). *Insect Species Conservation.* Cambridge University Press.

[bb65] Norvik, C., Westöö, C. K., Peruzzi, N., Lovric, G., van der Have, O., Mokso, R., Jeremiasen, I., Brunnström, H., Galambos, C., Bech, M. & Tran-Lundmark, K. (2020). *Am. J. Physiol. Lung Cell. Mol. Physiol.* **318**, L65–L75. 10.1152/ajplung.00103.2019 31596108

[bb66] Ollonen, J., Da Silva, F. O., Mahlow, K. & Di-Poï, N. (2018). *Front. Physiol.* **9**, 278. 10.3389/fphys.2018.00278 PMC5882870 29643813

[bb67] Paganin, D., Mayo, S. C., Gureyev, T. E., Miller, P. R. & Wilkins, S. W. (2002). *J. Microsc.* **206**, 33–40. 10.1046/j.1365-2818.2002.01010.x 12000561

[bb68] Pansani, T. R., Pobiner, B., Gueriau, P., Thoury, M., Tafforeau, P., Baranger, E., Vialou, V., Vialou, D., McSparron, C., de Castro, M. C., Dantas, M. A. T., Bertrand, L. & Pacheco, M. L. A. F. (2023). *Proc. R. Soc. B.* **290**, 20230316. 10.1098/rspb.2023.0316 PMC10336383 37434527

[bb69] Paukner, D., Wildenberg, G. A., Badalamente, G. S., Littlewood, P. B., Kronforst, M. R., Palmer, S. E. & Kasthuri, N. (2024). *Ecol. Evol.* **14**, e11137. 10.1002/ece3.11137 PMC10985371 38571794

[bb71] Prüßing, K., Voigt, A. & Schulz, J. B. (2013). *Mol. Neurodegeneration* **8**, 35. 10.1186/1750-1326-8-35 PMC4222597 24267573

[bb72] Quenot, L., Bohic, S. & Brun, E. (2022). *Appl. Sci.* **12**, 9539.

[bb73] Rawson, S. D., Maksimcuka, J., Withers, P. J. & Cartmell, S. H. (2020). *BMC Biol.* **18**, 21. 10.1186/s12915-020-0753-2 PMC7045626 32103752

[bb74] Rocha, C. F. D. & Vrcibradic, C. R. D. (1999). *Herpetol. J.* **9**, 43–53.

[bb75] Saccomano, M., Albers, J., Tromba, G., Dobrivojević Radmilović, M., Gajović, S., Alves, F. & Dullin, C. (2018). *J. Synchrotron Rad.* **25**, 1153–1161. 10.1107/S1600577518005489 29979177

[bb76] Sanchez, S., Ahlberg, P. E., Trinajstic, K. M., Mirone, A. & Tafforeau, P. (2012). *Microsc. Microanal.* **18**, 1095–1105. 10.1017/S1431927612001079 23026256

[bb77] Sasso Porto, D., Melo, G. A. R. & Almeida, E. A. B. (2016). *Rev. Bras. Entomol.* **60**, 109–113.

[bb78] Sena, G., Almeida, A. P., Braz, D., Nogueira, L. P., Soares, J., Azambuja, P., Gonzalez, M. S., Tromba, G. & Barroso, R. C. (2015). *Radiat. Phys. Chem.* **115**, 179–182.

[bb79] Sena, G., Fidalgo, G., Paiva, K., Barcelos, R., Nogueira, L. P., Colaço, M. V., Gonzalez, M. S., Azambuja, P., Colaço, G., da Silva, H. R., de Moura Meneses, A. A. & Barroso, R. C. (2022). *Biophys. Rev.* **14**, 625–633. 10.1007/s12551-022-00964-4 PMC9250583 35791381

[bb80] Serdeiro, M. T., Dias, T. D., de Lima, N. T. R., Barbosa-Filho, J. M., Belato, R. S., Santos-Mallet, J. R. & Maleck, M. (2023). *Tropical Med.* **8**, 440. 10.3390/tropicalmed8090440 PMC10534875 37755901

[bb81] Shin, S. (2021). *AAPPS Bull.* **31**, 21.

[bb82] Sire, J. Y. & Meunier, F. J. (1994). *Acta Zool.* **75**, 235–247.

[bb83] Snigirev, A., Snigireva, I., Kohn, V., Kuznetsov, S. & Schelokov, I. (1995). *Rev. Sci. Instrum.* **66**, 5486–5492.

[bb84] Soonwera, M., Moungthipmalai, T., Aungtikun, J. & Sittichok, S. (2022). *Heliyon* **8**, e09346. 10.1016/j.heliyon.2022.e09346 PMC9065628 35521510

[bb85] Souza-Neto, J. A., Powell, J. R. & Bonizzoni, M. (2019). *Infect. Genet. Evol.* **67**, 191–209. 10.1016/j.meegid.2018.11.009 PMC8135908 30465912

[bb86] Stein, K. & Prondvai, E. (2014). *Biol. Rev.* **89**, 24–47. 10.1111/brv.12041 23647662

[bb100] Suortti, P. & Thomlinson, W. (2003). *Phys. Med. Biol.* **48**, R1–R35. 10.1088/0031-9155/48/13/201 12884920

[bb87] Tafforeau, P., Boistel, R., Boller, E., Bravin, A., Brunet, M., Chaimanee, Y., Cloetens, P., Feist, M., Hoszowska, J., Jaeger, J. J., Kay, R. F., Lazzari, V., Marivaux, L., Nel, A., Nemoz, C., Thibault, X., Vignaud, P. & Zabler, S. (2006). *Appl. Phys. A* **83**, 195–202.

[bb88] Tao, S., He, C., Hao, X., Kuang, C. & Liu, X. (2021). *Appl. Sci.* **11**, 2971.

[bb89] Toh, Y. P., Dion, E. & Monteiro, A. (2021). *Methods Protoc.* **4**, 53. 10.3390/mps4030053 PMC8395752 34449688

[bb90] Töpperwien, M., Gradl, R., Keppeler, D., Vassholz, M., Meyer, A., Hessler, R., Achterhold, K., Gleich, B., Dierolf, M., Pfeiffer, F., Moser, T. & Salditt, T. (2018). *Sci. Rep.* **8**, 4922. 10.1038/s41598-018-23144-5 PMC5862924 29563553

[bb91] Vera Candioti, F., Goldberg, J., Akmentins, M. S., Nogueira Costa, P., Goulart Taucce, P. P. & Pombal, J. (2020). *Org. Divers. Evol.* **20**, 763–783.

[bb92] Vinauger, C., Lutz, E. K. & Riffell, J. A. (2014). *J. Exp. Biol.* **213**, 2321–2330. 10.1242/jeb.101279 PMC4081009 24737761

[bb93] Vommaro, M., Donato, S., Lo, L. K., Brandmayr, P. & Giglio, A. (2022). *J. Anat.* **242**, 510–524. 10.1111/joa.13796 PMC9919503 36417320

[bb94] Westfahl, H. Jr, Tolentino, H. C., Meneau, F., Archilha, N., Neto, N. M. S., Lin, L., Sanfelici, L., Meyer, B. M., Polli, J. M. & Miqueles, E. (2018). *Microsc. Microanal.* **24**, 176–179.

[bb95] Westneat, M. W., Socha, J. J. & Lee, W. K. (2008). *Annu. Rev. Physiol.* **70**, 119–142. 10.1146/annurev.physiol.70.113006.100434 18271748

[bb96] Wilkins, S. W., Nesterets, Y. I., Gureyev, T. E., Mayo, S. C., Pogany, A.& Stevenson, A. W. (2014). *Philos. Trans. R. Soc. A.* **372**, 20130021. 10.1098/rsta.2013.0021 24470408

[bb97] Wu, J. S. & Luo, L. (2006). *Nat. Protoc.* **1**, 2110–2115. 10.1038/nprot.2006.336 17487202

[bb98] Zehbe, R., Haibel, A., Riesemeier, H., Gross, U., Kirkpatrick, C. J., Schubert, H. & Brochhausen, C. (2010). *J. R. Soc. Interface.* **7**, 49–59. 10.1098/rsif.2008.0539 PMC2839371 19324670

[bb99] Zhou, S. A. & Brahme, A. (2008). *Phys. Med.* **24**, 129–148. 10.1016/j.ejmp.2008.05.006 18602852

